# Understanding Adolescents’ Need Support, Need Satisfaction, and Health-Related Outcomes: A Self-Determination Health Behavior Perspective

**DOI:** 10.3390/ijerph17010104

**Published:** 2019-12-22

**Authors:** Changzhou Chen, Tao Zhang, Xiangli Gu, Joonyoung Lee, Sutang Ren, Hongying Wang

**Affiliations:** 1 School of Physical Education and Training, Shanghai University of Sport, Shanghai 200438, China; Changzhou.Chen@unt.edu; 2 Department of Kinesiology, Health Promotion and Recreation, University of North Texas, Denton, TX 76262, USA; joonyounglee@my.unt.edu; 3 Department of Kinesiology, University of Texas at Arlington, Arlington, TX 76019, USA; Xiangli.Gu@uta.edu; 4 Putuo Education College, Shanghai 20061, China; Sutang.Ren@gmail.com; 5 School of Leisure Sport, Shanghai University of Sport, Shanghai 200438, China

**Keywords:** self-determination theory, basic psychological needs, physical activity, health-related quality of life, adolescents

## Abstract

School physical education (PE) as an important social context can promote adolescents’ physical health and contributes to their mental health. Guided by the self-determination health behavior model, the study aimed to examine a structural mediation model to investigate the relationships among perceived need support from PE teachers, psychological need satisfaction, and adolescents’ health-related outcomes. Participants were 300 adolescents (M_age_ = 14.48; 50.3% girls) recruited from five middle schools in Shanghai, China. They completed previously validated questionnaires assessing their perceived need support from PE teachers, psychological need satisfaction, leisure time physical activity (LTPA), and health-related quality of life (HRQOL). The hypothesized model demonstrated a good fit (χ^2^/df = 3.4, *p* < 0.01; Root Mean Square Error of Approximation (RMSEA) = 0.09; Standardized Root Mean Square Residual (SRMR) = 0.06; Bentler–Bonett Nonnormed Fit Index (NFI)= 0.92; Comparative Fit Index (CFI) = 0.94; 90% Confidence Interval (CI) [0.07, 0.11]). The findings indicated that three basic psychological needs can be satisfied by perceived need support from PE teachers, and psychological need satisfaction was positively associated with health-related outcomes such as LTPA and HRQOL. In addition, psychological need satisfaction mediated the relationship between perceived need support from PE teachers and health-related outcomes such as LTPA and HRQOL in the present study. The findings supported the theoretical tenets of the self-determination health behavior model and its generalizability among Chinese adolescent students.

## 1. Introduction

It is well documented that moderate-to-vigorous physical activity (MVPA) is associated with physical and psychological health benefits [1]. According to the 2018 Physical Activity Guidelines, adolescents should engage in at least 60 min of MVPA daily [2]. In fact, among Chinese adolescents, only 29.9% of them engage in the recommended 60 min MVPA per day, more than half of them involve in screen-based entertainments (e.g., playing video or computer games) for three or more hours per day [3,4]. In addition, research noted that the prevalence of physical inactivity and sedentary behaviors increased with age during adolescence, and obesity in this population had tripled in the past three decades among Chinese adolescents [5,6]. 

Given that adolescence is a critical period for establishing an independent healthy lifestyle, the prevalence of physical inactivity and related health problems should be addressed [7]. School physical education (PE) as an important social context not only promotes adolescents’ physically active lifestyle but can also enhance other health outcomes, such as improving their health-related quality of life (HRQOL) [8,9,10]. To promote and maintain a physically active lifestyle among school-aged adolescents, it is essential to identify the relationships between social environmental factors, psychological factors, and health-related outcomes [11]. According to self-determination theory (SDT), health-related behaviors can be promoted by satisfying individuals’ basic psychological needs by creating a need-supportive social environment [12].

As a general theory of motivation, SDT has been highlighted as the relevant psychological energy to initiating and maintaining new health-related behaviors [12]. When three basic psychological needs are satisfied in a given domain, the individuals will move toward more autonomous self-regulation around the behaviors and self-determined motivation. SDT has also shown that need-supportive environments (such as the degree of autonomy support, competence support, and relatedness support) are more likely to motivate an individual to self-regulate and initiate new health-related behaviors [13,14]. Recently, SDT has been used in studies of health context addressing health behavior change, healthcare environment, and interventions [15,16]. The self-determination health behavior model has been well-established and used to explicate how SDT constructs interrelate and predict indices of mental and physical health [13]. 

According to the self-determination health behavior model, three basic psychological needs (i.e., autonomy, competence, and relatedness) are central concepts that explain the process of internalization and integration of health behavior change. The concept of autonomy reflects the feeling of being the origin of one’s own behaviors. Competence is described as the feeling of achieving desired outcomes, and relatedness is defined as the feeling of being understood and cared for by others. Once these three basic psychological needs are satisfied in a given domain context, the individuals will move toward more autonomous self-regulation to foster optimal physical, psychological, and social functioning [12,17].

The self-determination health behavior model suggests the contextual-level social environmental factors (autonomy supportive climate, personality differences in autonomy, and extrinsic life aspirations) that may be the essential nutriments to optimize three basic psychological needs. In the context of PE, some intervention research suggested that the PE teacher–student relationship as an influential medium and vehicle of change, and PE teachers’ interpersonal style for specific behavior strategies may support their students’ basic psychological needs [18,19]. Research evidence also found that the more autonomous regulation an individual receives, the greater health behavior an individual will perform (i.e., effort, concentration, confidence, leisure-time physical activity [LTPA], etc.) [20].

As proposed by the self-determination health behavior model, the satisfaction of these basic psychological needs may lead to better HRQOL and promote PA participation. Evidence from previous studies has confirmed that a high perceived need satisfaction positively predicted the high level of PA [21]. As a part of the overall quality of life, HRQOL was increasingly being used to measure individuals’ perception of their physical and mental health. HRQOL is a comprehensive and multidimensional construct of pediatric population health where adolescent health outcomes include physical, social, emotional, and school functioning [22]. Morgan et al. testing the HRQOL of obese adolescents have concluded that obesity in adolescence was related to poor health [23]. Bize and colleagues found that the individuals’ HRQOL can be improved by taking more physical activities [24]. Recent research also suggested that HRQOL was influenced by some motivational factors (i.e., perceived competence, autonomy support) among adolescents [25,26].

**Figure 1:**
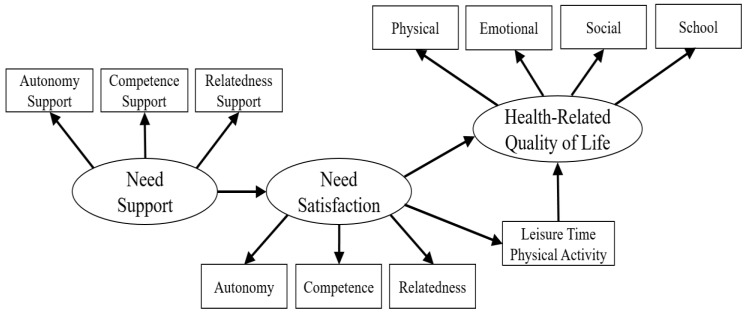
Hypothesized Model of the Variables. Note. Circles represent latent variables, and squares represent observed variables.

However, the underlying mechanisms regarding the indirect effects (mediating effects) of social environmental factors on physical and psychosocial health outcomes through psychological need satisfaction (personal psychological factor) has not been established. Guided by the self-determination health behavior model, this study aimed to test a structural mediation model (see Figure 1) in order to understand how perceived need support from PE teachers (i.e., autonomy support, competence support, and relatedness support) may indirectly influence health-related outcomes (i.e., LTPA and HRQOL) through psychological need satisfaction (i.e., competence, autonomy, and relatedness). Specifically, it was hypothesized that (1) perceived need support from PE teachers would be positively related to students’ psychological need satisfaction; (2) students’ psychological need satisfaction would be positively associated with their health-related outcomes such as LTPA and HRQOL; (3) students’ psychological need satisfaction would mediate the relationship between perceived need support from PE teachers and health-related outcomes such as LTPA and HRQOL in the present study.

## 2. Methods

### 2.1. Participants

Participants in this study were recruited from five public middle schools at the same school district of Shanghai, China. Each school has similar student enrollments and school physical activity environments, such as spaces, equipment, and facilities. We randomly recruited 60 eighth grade students at each school, and there were 300 adolescents (50.3% girls, M _age_ = 14.48 years, SD = 0.53) in our final sample. Permission to conduct this study was granted by the university’s institutional review board (IRB) with the approval number #17536, and the study was also approved by the school district, school principals, and PE teachers prior to data collection. In addition, parental informed consent and child assent forms were obtained from all participants before data collection.

### 2.2. Measures

#### 2.2.1. Perceived Need Support

Three scales were used to measure adolescents’ perceived need support from PE teachers consisting of perceived autonomy support, perceived competence support, and perceived relatedness support. Six-item physical education-modified health climate questionnaire (HCCQ) assessed students’ perceived autonomy support [27]. A sample item is, “*We feel that the PE teacher provides us with choices and options*”. Moreover, four items measured students’ perceived competence support [28]. An example item is, “*The PE teacher helps us to improve in class*”. Finally, five items measured students’ perceived relatedness support. “*The PE teacher has respect for us*” and “*The PE teacher supports us*” are two example items. All perceived need support was rated on a seven-point scale from 1 (*strongly disagree*) to 7 (*strongly agree*). These scales have all demonstrated acceptable reliability and validity in previous studies [28], and have indicated satisfactory internal reliability coefficients in this study.

#### 2.2.2. Psychological Need Satisfaction

Participants’ psychological need satisfaction toward PE was assessed using previously validated scales, including perceived autonomy, competence, and relatedness. Students’ perceived autonomy was measured using a six-item scale devised by Standage and colleagues [28] with an example item “*I have some choice in what I want to do in PE class*”. To assess the degree of the students’ perceived competence, five items from the perceived competence of the Intrinsic Motivation Inventory were used [29]. A sample item is, “*When I have participated in PE for a while, I feel pretty competent*”. Additionally, the modified five-item scale of the Need for Relatedness Scale [30] for PE was used to measure students’ perceived relatedness. The psychological need satisfaction scale was rated on a seven-point scale from 1 (*strongly disagree*) to 7 (*strongly agree*). These scales demonstrated acceptable internal reliability in this study.

#### 2.2.3. Health-Related Quality of Life (HRQOL).

The Pediatric Quality of Life Inventory (PedsQL^TM^ 4.0, Varni et al., 2001) [31] was used for assessing participants’ HRQOL, including physical functioning (8 items; e.g., *It’s hard for me to do sports or activities*); emotional functioning (5 items; e.g., *I feel afraid or scared*); social functioning (5 items; e.g., *It’s hard to keep up when I play with other kids*); and school functioning (5 items; e.g., *It’s hard to pay attention in class*). All items followed the stem “*How much of a problem has this been for you in the past 7 days*”. Items were scored using a five-point reverse-scale ranging from 0 (*never a problem*) to 4 (*almost always a problem),* and were converted into a 100 linear scale (*0 = 100, 1 = 75, 2 = 50, 3 = 25, 4 = 0*). A higher score indicates better quality of life. The scale demonstrated acceptable reliability in this study.

#### 2.2.4. Leisure-Time Physical Activity (LTPA)

The Godin Leisure-time Exercise Questionnaire was used to assess participants’ level of strenuous, moderate, and light PA during a typical 7-day period [32]. Participants were asked to indicate the amount of PA they have engaged in the past week. For instance, *in the past week, how many 15 min of strenuous exercise activity have you participated in?* (Which is defined as “it makes my heart beat quickly, and makes me sweat”, such as running, football, basketball, vigorous swimming). The total weekly leisure-time PA score was calculated based on the equation: (9 × Strenuous) + (5 × Moderate) + (3 × Light), and the total score is represented as students’ LTPA level in this study. 

### 2.3. Research Design and Procedure

A cross-sectional research design was used in this study. The survey data including students’ perceived need support, psychological need satisfaction, HRQOL and LTPA, were administered in the middle of the fall semester in 2018. The instrument was translated from English to Chinese and back-translated from Chinese to English by a team of three independent bilingual translators. To ensure the validity of the questionnaire, the original English and back-translation versions were compared, and all discrepancies were resolved and accepted by all three bilingual translators. In addition, the Chinese versions of all scales were sent to the panel members, including five PE teachers and three pedagogy professors for final review and approval in order to get acceptable content-related validity. Before the questionnaires were administered, a training session for research assistants was conducted on how to administer the questionnaires appropriately as per the IRB approval process. Questionnaires were administered only by trained research assistants, who were also available on site to answer questions. On average, it took each participant about 20–25 min to complete the questionnaires.

### 2.4. Data Analyses

The data were analyzed using the Statistical Package of the Social Sciences (SPSS 25.0, IBM Corp., Armonk, NY, USA) and AMOS 25.0. Specifically, descriptive statistics and internal consistency estimates were calculated in SPSS 25.0 for all variables, and Pearson’s product-moment correlation was also computed to explain the bivariate relationships among perceived need support (i.e., perceived autonomy support, competence support, relatedness support), psychological need satisfaction (i.e., autonomy, competence, relatedness), and health-related outcomes (i.e., HRQOL and LTPA). The Structure Equation Modeling (SEM) was constructed to test the hypothesized mediation model (see Figure 1). Before structuring the whole model, a confirmatory factor analysis (CFA) was conducted to test measurement models. Then, according to Figure 1, the whole mediation model was structured to test the mediational role of psychological need satisfaction in the relationship between perceived need support and health-related outcomes. In addition, the bootstrapping procedure was conducted to assess the statistical significance of the indirect effects (mediation model) by calculating 95% confidence intervals [33].

The following various indices of fit were examined to evaluate the adequate fit of the model to the data: the Chi-Squared test (χ^2^), Root Mean Square Error of Approximation (RMSEA), Standardized Root Mean Square Residual (SRMR), Bentler–Bonett Nonnormed Fit Index (NFI), and Comparative Fit Index (CFI) [34,35]. Specifically, the χ^2^ test tests whether there is a statistically significant difference between model and sample data and degrees of freedom (*df*) for each estimated model. Given that χ^2^ can be heavily influenced by sample size, a χ^2^/df ratio between 2 and 5 has often been employed. The model fits were also determined using other goodness-of-fit indices: (a) acceptable model fit when RMSEA is less than 0.10 and SRMR is less than 0.08; (b) reasonable model fit when both NFI and CFI is greater than 0.90., and values greater than 0.95 are typically considered an excellent fit [34,35].

## 3. Results

### 3.1. Descriptive Analysis, Scale Reliability, and Correlation

Table 1 shows the means, standard deviations, alpha coefficients, and Pearson’s product-moment correlations among the study variables assessed in the present study. The mean scores for all study variables were generally above the moderate range, indicating that participants had positive perceptions of teachers’ support, psychological need satisfaction, LTPA, and HRQOL. Further, the reliability coefficient of self-reported measures had demonstrated acceptable internal consistency, exceeding the criterion of 0.70 [36].

As demonstrated by the pattern of correlations presented in Table 1, six subscales of both perceived need support and psychological need satisfaction were positively related to HRQOL (r values ranging from 0.29 to 0.52, *p* < 0.01). In addition, LTPA was significantly associated with HRQOL, the three subscales of psychological need satisfaction, perceived autonomy support, and competence support (r values ranging from 0.15 to 0.31, *p* < 0.05), except for perceived relatedness support. 

### 3.2. Testing Hypothesized Structural Model

Before testing the hypothesized structural model, a CFA was first constructed to estimate the latent variables, such as perceived need support and psychological need satisfaction. The results of the perceived need support model (χ^2^/df = 5.2, *p* < 0.01; RMSEA = 0.11; SRMR = 0.06; NFI = 0.91; CFI = 0.92; 90% CI [0.11, 0.13]) and the psychological need satisfaction model (χ^2^/df = 4.2, *p* < 0.01; RMSEA = 0.10; SRMR = 0.07; NFI = 0.88; CFI = 0.90; 90% CI [0.09, 0.11]) did not provide good fits to the data. Using the modification indices, the goodness-of-fit indices of perceived need support model (χ^2^/df = 2.5, *p* < 0.01; RMSEA = 0.07; SRMR = 0.03; NFI = 0.96; CFI = 0.97; 90% CI [0.06, 0.09]) and psychological need satisfaction model (χ^2^/df = 2.3, *p* < 0.01; RMSEA = 0.07; SRMR = 0.06; NFI = 0.94; CFI = 0.96; 90% CI [0.06, 0.08]) provided good fits to the data after the error covariance. Moreover, all the standardized factor loadings of the observed variables on their respective latent variables were higher than 0.40 and statistically significant, indicating that each latent variable was appropriately supported by validity constructs of the measurement models [37].

Based on validity constructs of the measurement models, the hypothesized structural model of the study variables was tested. First, we assessed the fit of the model by specifying that perceived need support would be directly associated with HRQOL and LTPA, but with no path specified connecting perceived need support to psychological need satisfaction. However, the goodness-of-fit indices of the first model (not shown) indicated that the model did not fit the data (χ^2^/df = 7.6, *p* < 0.01; RMSEA = 0.15; SRMR = 0.23; NFI = 0.82; CFI = 0.84; 90% CI [0.13, 0.16]). Second, we added the direct path from perceived need support to mediator (psychological need satisfaction), and the results of the second model suggested a well-fitting model (χ^2^/df = 3.4, *p* < 0.01; RMSEA = 0.09; SRMR = 0.06; NFI = 0.92; CFI = 0.94; 90% CI [0.07, 0.11]). As suggested in previous studies [38,39], the second model fits better than the first model, where the mediation of the psychological need satisfaction model can be supported.

**Figure 2:**
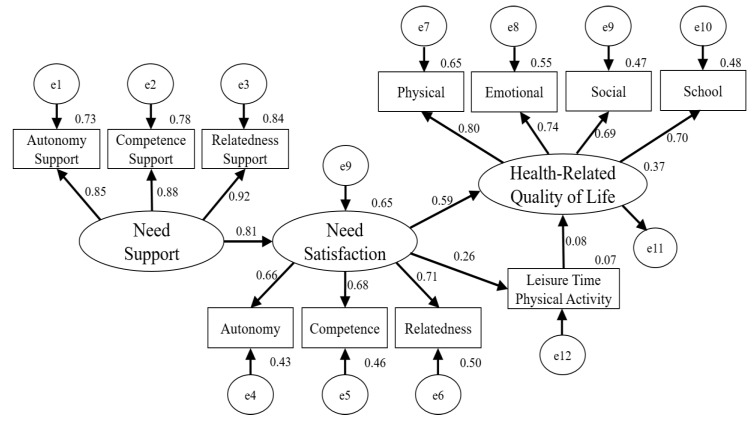
Final Structural Model of the Study Variables. Note. Circles represent latent variables, and squares represent observed variables. All paths are significant at *p* < 0.05.

The standardized direct effects of the final model (see Figure 2) showed that perceived need support was a strong predictor of psychological need satisfaction (*β* = 0.81, *p* < 0.05), and path coefficients from psychological need satisfaction to HRQOL (*β* = 0.59, *p* < 0.01) and LTPA (*β* = 0.26, *p* < 0.01) were also significant in this model. However, LTPA was not a significant predictor of HRQOL in this study. The standardized indirect effects showed that perceived need support positively predicted autonomy (*β* = 0.53, *p* < 0.01), competence (*β* = 0.55, *p* < 0.01), and relatedness (*β* = 0.57, *p* < 0.01). Moreover, the indirect path from perceived needs support to LTPA (*β* = 0.21, *p* < 0.01) and HRQOL (*β* = 0.49, *p* < 0.01) were significant in this model. After controlling for all covariates, the bootstrapping procedure was conducted to assess the statistical significance of the indirect effects (mediation model). The results showed 95% CI [0.36, 0.60] for HRQOL and 95% CI [0.11, 0.30] for LTPA, respectively. The squared multiple correlations of the final model revealed that perceived need support accounted for 65% of the variance in psychological need satisfaction, while perceived need satisfaction accounted for 37% of the variance in HRQOL and 7% of the variance in LTPA, respectively.

## 4. Discussion

Guided by the self-determination health behavior model, this study aimed to test a structural mediation model (see Figure 1) in order to understand how perceived need support from PE teachers (i.e., autonomy support, competence support, and relatedness support) may indirectly influence health-related outcomes (i.e., LTPA and HRQOL) through psychological need satisfaction (i.e., competence, autonomy, and relatedness). Based on validity constructs of the measurement models, the hypothesized structural model of study variables was tested, and the results have confirmed an acceptable fit to the data in the present study. Further, direct and indirect effects of students’ perceived need support from PE teachers on their LTPA and HRQOL were also tested, and the results suggested that psychological need satisfaction plays a significant mediating role between perceived need support and health-related outcomes (i.e., LTPA and HRQOL) in this study. 

One of the most important findings was that basic psychological needs were significant predictors of adolescents’ health-related outcomes, such as LTPA and HRQOL, in this study. Assessing PA and HRQOL have been highlighted by many researchers in pediatric public health to reflect their physical and mental health [9]. Research has found that adolescents’ physical and mental health still faced serious issues [40]. There is some evidence documenting the significant relationship between basic psychological needs and LTPA [21]. Other researchers have found that satisfying these basic psychological needs in the PE context can promote overall HRQOL and more physical activities in middle school students [21,22]. In addition, Gu and colleagues suggested that PA was positively associated with HRQOL among middle school students [9,25]. However, the results of this study demonstrated that LTPA was not a significant predictor of adolescents’ HRQOL. Consistent with the tenets of SDT, the three basic psychological needs are considered to be necessary nutriments to predict PA, healthy functioning, and well-being [13,22]. In light of previous studies, the findings of this study also revealed the important role of adolescents’ psychological needs on their health-related outcomes.

In addition, previous studies have shown that these three basic psychological needs can significantly impact individuals’ behavior outcomes, in which autonomy should be met first, then competence, and finally relatedness [21,22]. In the present study, the findings identified that the strongest predictor of students’ LTPA was competence, followed by autonomy, and finally relatedness, which differs from the findings of Standage et al. [22] but confirms the findings of Ntoumanis et al. [41]. The reason for this inconsistency in this study may be related to the study participants being middle school students in China, who are experiencing the challenges of adolescence, valuing personal competence to gain recognition and status among their peers. According to the adolescents’ various psychological needs, the findings of this study may have implications for practitioners to design effective health promotion programs in PE classes to improve adolescents’ physical and mental health.

In accordance with the study hypotheses as well as findings from previous studies [22,26,42], our findings indicated that perceived need support from PE teachers was positively related to students’ psychological need satisfaction. The results suggested that social environments (autonomy support, competence support, and relatedness support) created by PE teachers can play important roles with respect to students’ three basic psychological needs. As proposed by the SDT, teachers’ interpersonal style for specific behavior strategies may support their students’ three basic psychological needs in the PE context [43]. Research evidence suggested that the autonomy-supportive environment provided by the PE teacher is characterized by acknowledging the students’ perspective so as to give a rationale, choice, and promote self-endorsed reasons for acting [44,45]. Likewise, competence support can be facilitated if PE teachers provide an overview and communicate expectations, give constructive feedback, and provide self-referenced standards and self-improvement [11]. In addition, establishing peer-learning groups, changing the strategy to form groups, structuring opportunities for positive peer interaction, and being close and friendly with students are other means that PE teachers can adopt to promote students’ perceptions of relatedness [43,46]. Collectively, the findings of this study confirm those of previous PE-related research that showed these students’ three basic psychological needs could be fostered when PE teachers employ these strategies effectively. 

Further, after testing indirect effects of students’ perceived need support from PE teachers on their health-related outcomes such as LTPA and HRQOL, the results showed that psychological need satisfaction plays a mediating role. The results of this study are consistent with the findings of Standage and colleagues, confirming the significant roles of these three basic psychological needs [47]. However, other studies have proven that parts of the three basic psychological needs have substantial effects when the three basic psychological needs are used as mediating variables. For example, Reinboth and colleagues found that in the relationship between social context and students’ general well-being, only autonomy and competence had a substantial mediating effect [48]. The reason might be due to differences in the cultural background in our respective study samples. In China, PE teachers always want students to acquire more physical fitness and motor skills to cope with the standardized examination, while ignoring the students’ relatedness need. This can also lead to a typical social phenomenon in China, where students often like sports but do not like their PE classes [6]. 

The findings from this study suggest that school adolescents’ health-related outcomes can be promoted by satisfying their three basic psychological needs by creating a need-supportive social environment in schools. This study not only provided us with a clear perspective to understand health behavior change among school adolescents but also provided helpful suggestions for school PE teachers and school health advocates, such as school psychologists or nurses, to improve students’ health-related outcomes in schools. Firstly, school health advocates and PE teachers should design different teaching strategies to address the basic psychological needs of a diversified student population, given that only when students’ psychological needs are all adequately met can their health and wellness be truly promoted. Secondly, school health advocates and PE teachers should create a need-supportive environment (such as autonomy support, competence support, and relatedness support) to motivate students to self-regulate and initiate positive health-related outcomes. Lastly, school principals and other leaders should pay more attention to PE classes and strengthen the cooperation with governments, school health advocates, and PE teachers to promote the professional development of PE teachers.

There are also a few limitations to this study. The first limitation of this study was the use of self-reported LTPA measure rather than the use of an objective measure, which may increase the potential for personal bias in recall. Future investigation should use an objective measure such as accelerometers or pedometers to assess students’ LTPA. Another limitation was the cross-sectional research design, which means the causal conclusions could not be inferred by using SEM analyses in this study. Further investigations are warranted to adopt experimental research designs or longitudinal research designs to examine the causal relationships among study variables. Finally, due to the complexity of middle school students’ psychological state, this study focused on the impact of external social environmental factors on students’ psychological need satisfaction but ignored the role of the students’ personality traits. Future research is needed to address students’ personality traits in order to have a better understanding of students’ psychological state.

## 5. Conclusions

In summary, this study tested a hypothesized structural mediation model based on the self-determination health behavior model, which enhances our understanding of the relations between perceived need support, psychological need satisfaction, LTPA, and HRQOL among middle school students in China. The results of this study indicated that students with higher perceived need support from PE teachers reported greater satisfaction of their three basic psychological needs, and better levels of health-related outcomes, such as LTPA and HRQOL. Psychological need satisfaction mediated the relationship between perceived need support from PE teachers and health-related outcomes such as LTPA and HRQOL in the present study. The findings support the theoretical tenets of the self-determination health behavior model and its generalizability among Chinese adolescent students.

## Figures and Tables

**Table 1:** Descriptive Statistics, Internal Consistency, and Correlations among the Study Variables (N = 300).

Subscale	1	2	3	4	5	6	7	8
1. Autonomy support	(0.92)							
2. Competence support	0.73 **	(0.82)						
3. Relatedness support	0.79 **	0.82 **	(0.93)					
4. Autonomy	0.52 **	0.55 **	0.55 **	(0.73)				
5. Competence	0.48 **	0.48 **	0.42 **	0.39 **	(0.83)			
6. Relatedness	0.53 **	0.53 **	0.52 **	0.46 **	0.48 **	(0.94)		
7. HRQOL	0.38 **	0.34 **	0.34 **	0.29 **	0.52 **	0.39 **	(0.92)	
8. LTPA	0.19 **	0.15 *	0.10	0.18**	0.31 **	0.15 *	0.22 **	-
M	5.58	5.79	5.99	4.83	5.11	5.76	80.88	63.81
SD	1.26	1.00	1.06	1.04	1.19	1.27	14.07	30.03

Note: Cronbach’s alpha for the study variables are provided along the diagonal; HRQOL = Health-Related Quality of Life; LTPA = Leisure Time Physical Activity; M = mean; SD = standard deviation; ** *p* < 0.01; * *p* < 0.05.
